# Supplementary material for: Acceptance and compliance with micronutrient powder (MNP) among children aged 6–23 months in northern Nigeria
Source: PLOS Glob Public Health. 2022 Oct 17;2(10):e0000961. doi: 10.1371/journal.pgph.0000961 (PMC10022258; doi:10.1371/journal.pgph.0000961)
Supplement: S1 Dataset — (ZIP) [file pgph.0000961.s008.zip › S8_Dataset/Direct Observations.zip!/Direct Observations/220615_K_J_SA_1_DO.pdf]

1.1.1 Instrument F –Direct Observation Form

<<**Instrument F Direct Observation Form**>>

PI Name: PragyaMathema,Stephen Kodish
Study Title: Formative research to develop an infant and young child feeding (IYCF) intervention with micronutrient powder (MNP) for children aged 6 – 23 months
PI Version/Date: Version1.0/January 24, 2015

Date: 22/06/15Location:Tanza Babba/ Jega

Data Collector: Samira Adamu

Main goals of this direct observation are to answer the following questions:

1. In this household, what would be the best way(s) to give nutritional supplements to young children? Why?

2. In this household, describe the food sharing that you observe throughout the meal. Are there any predictable patterns?

3. In this household, who is favored and how is this favoritism expressed during meal preparation and eating?

Household Composition Table (Fill out as you do data collection)

INITIALS	SEX (M/F)	AGE (estimated)	Relationship to household	Description (not necessary)
BGS	M	7months	Youngest child	Baby boy wearing a green sweater
MB	F	20years	Mother of the child(daughter-in-law)	Medium height wearing a pink and a black vest
DL1	F	25years	Wife of the first son	Medium height wearing a striped shirt with green veil
DL2	F	22years	Wife of first son	Short, wearing a maroon bra with brown dotted scarf
ML	F	55years	Mother-in-law, grandmother(child)	Dark and tall with green wrapper and black veil
MML1	F	45years	Mother-in-law (wife of the uncle)	Slim with green and red wrapper with peach veil
MDL1	F	20years	Uncle's daughter-in-law	Fair complexioned with blue and yellow wrapper and pink scarf
MDL2	F	16years	Uncle's daughter in-law	Dark complexioned with yellow and orange wrapper and orange scarf
BGM	F	10months	cousin	Wearing light green shirt and two round

				ear-rings
BB4	F	4years	5th child	Wearing a striped shirt and a bit fair and plump
BB3	F		4th child	Dark wearing white and peach shirt with black skirts
BB2	F		3rd child	Wearing an orange vest with brown skirt
BB1	F	10years	1st child	Wearing red and stripped shirt with tribal mark
FL	M	70years	Father-in-law	Slim and fair skinned complexioned old man

PARTICIPANT ID: __________

TIME	ACTOR INITIALS	RECIPIENT INITIALS	WHAT HAPPENED	FOOD	Reactivity
8:40	MB	BGS	Backing the baby while standing		
8:45	MB	BGS	Backing the baby while standing		
	MB		Pounding grains for processing to remove shaft	maize	
	MB&DL		Pounding grains processing to remove shaft	maize	
8:50	MB	BGS	Backing the baby while standing		
	MB		Still pounding grains for processing to remove shaft	maize	
	DL		Processing grains to remove shaft	maize	
8:55	MB	BGS	Feeding the child breast milk while standing	Breast milk	
9:00	MB	BGS	Feeding the child breast milk while standing	Breast milk	
9:05	MB	BGS	Feeding the child breast milk while standing	Breast milk	
	MB		Still feeding the baby and pounding maize	Breast milk	
9:10	MB	BGS	Still feeding the baby and pounding maize	Breast milk	

TIME	ACTOR INITIALS	RECIPIENT INITIALS	WHAT HAPPENED	FOOD	Reactivity
	MDL1		Pounding maize to process by removing shaft	Maize	
	MDL2		Pounding maize to process to remove shaft	Maize	
	DL1		Pounding maize to process to process shaft.	Maize	
9:15	BGS		The child is sleeping on the mothers back		
	MB	BGS	Backing the baby and pounding maize	Maize	
	DL1<br>DL2	BGM	Both are pounding maize to process by removing shaft.	Maize	
	MDL1		Backing the baby and pounding the maize	Maize	
	MDL2		Processing maize to remove shaft		
9:20	DL2		Sieving grounded flour to remove the coarse grains and obtain fine flour.	Maiwa	
	BGS		The child is sleeping on the mat		
	ML		Washing her wrapper		
9:25	BGS		The child is sleeping on the mat		
	DL2		Sieving grounded flour to remove the coarse grain and obtain fine flour.	Maiwa	
	MDL1&<br>MDL2		Both pounding maize to flour.		
9:30	BGS		The child is sleeping on the mat		
9:35	BGS		The child is sleeping		
	MB		She is washing a pot		
9:40	BGS		The child is still sleeping on the mat.		
	MB		Washing the processed maize to sieve out shaft	Maize	

TIME	ACTOR INITIALS	RECIPIENT INITIALS	WHAT HAPPENED	FOOD	Reactivity
			with calabash.		
	MDL1& MDL2		Still pounding the maize, a process to remove the shaft	Maize	
9:45	BGS		The child is still sleeping on the mat.		
	MB		Draining the maize in a sieve.	Maize	
	MB	BGS	Backing the baby		
9:50	MB	BGS	Backing the baby		
	MB		Selling tamarind to a girl from the neighborhood	Tarmarind	
9:55	DL2		Sieving fine grounded flour	Maize	
	BGS		The child is still sleeping on the mat		
	MB	BGS	Backing the baby		
10:00	DL2		Sieving grounded maize to obtain fine flour.	maiwa	
	MB	BGS	Backing the baby		
	BGS		The child is sleeping on the mat.		
10:05	BGS		The child wakes up and started crying; the mother came and backs the child.		
	MB	BGS	The mother is still backing the child		
	DL2		Still grinding the cereal into flour	Maiwa	
10:10	MB		Backing the baby		
	MB		Sieving grounded flour to obtain fine flour.	Maiwa	
	BB3	BB1	Giving the other child rice in a transparent polythene bag to eat.	Rice	
	BB3	BB2&BB1	Sharing rice with the other	rice	

TIME	ACTOR INITIALS	RECIPIENT INITIALS	WHAT HAPPENED	FOOD	Reactivity
			children from the transparent polythene bag.		
10:15	MB	BGS	Backing the baby		
	DL2		Pounding the coarse grains obtained after sieving maiwa flour	Maiwa	
10:20	MB		Still backing the baby		
	DL2		Pounding the remaining maiwa cereal	Maiwa	
10:25	MB	BGS	Breastfeeding the baby	Breast milk	
10:30	MB	BGS	Breastfeeding the baby	Breast milk	
10:35	MB	BGS	Making the child urinate by placing him in between her two legs		
	ML		Playing with the child		
10:40			Breastfeeding the child	Breast milk	
10:45	BGS		The child is crying, the mother carried the child and started feeding the child.	Breast milk	
10:50	MB	BGS	Breastfeeding the baby	Breast milk	
	BGS		Playing with the other children		
10:55	MB		Processing moringa by removing the leaves from the stick	Moringa(dakaule)	
	DL2		Also processing the moringa		
	BGS		Playing on the floor with moringa sticks		
11:00	MB		Processing moringa by removing the leaves from the stick	Moringa	
	DL2		Also processing the		

TIME	ACTOR INITIALS	RECIPIENT INITIALS	WHAT HAPPENED	FOOD	Reactivity
			moringa by removing leaves from the stick.		
	BGS		Playing with the moringa sticks and putting it in his mouth		
11:05	BGS		Playing with other children		
	MB		Still processing moringa by removing leaves from the stick.	Moringa	
	DL2		Still processing moringa by removing leaves from the stick.	Moringa	
11:10	MB	BGS	Breastfeeding the child	Breast milk	
	DL2&MB		Both are still processing moringa	Moringa	
11:15	BGS		Playing with his siblings		
	MB&DL2		Still processing moringa	Moringa	
11:20	BB1	BGS	Carrying the child on her laps		
11:25	BGS		Crawling around the house		
11:30	BGS		Still playing with the moringa sticks	Moringa	
	MB&DL2		Processing of moringa by removing the leaves from the sticks.		
11:35	MB	BGS	Breastfeeding the child	Breast milk	
11:40	MB	BGS	Breastfeeding the child	Breast milk	
	MDL2&MB		Still Processing the moringa leaves to remove leaves from sticks		
11:45	BGS		Crawling around the house and eating raw dankaule (moringa)	Moringa	
	MML	BGS	Carrying the child to her		

TIME	ACTOR INITIALS	RECIPIENT INITIALS	WHAT HAPPENED	FOOD	Reactivity
11:50			side of apartment in the house		
11:55	MML	BGS	Playing with the child by her side		
12:00	BGS		Playing with stainless bowl		
	MB		Sitting and watching the baby play with the stainless bowl.		
12:05	BGS		Still playing with bowl alone,		
12:10	BGS		Still playing with bowl alone		
12:15	BGS<br>MB		Still playing with bowl alone as the mother watches him.		
12:20	BGS		The child is playing with his hands		
12:25	MB	BGS	She is breastfeeding the child while sitting on a stool.	Breast milk	
12:30	BGS		Stooling on the floor		
	MB		Holding the child as he stools on the floor		
12:45	MB	BGS	Bathing the baby inside the bathroom.		
12:50	MB	BGS	Bathing the baby inside the bathroom		
12:55	MB	BGS	Wearing pant for the child		
1:00	MB	BGS	Sitting on the mat and breastfeeding the baby	Breast milk	
1:05	MB	BGS	Lying on the mat with the baby and breastfeeding the baby	Breast milk	
1:10	MB	BGS	Lying on the mat with the and breastfeeding the baby.	Breast milk	

TIME	ACTOR INITIALS	RECIPIENT INITIALS	WHAT HAPPENED	FOOD	Reactivity
	BGS		Sleeping on the mat alone		
1:15	BGS		Sleeping on the mat		
1:20	BGS		Sleeping on the mat alone.		
1:25	BGS		Sleeping on the mat alone.		
	MB		Processing bitter leaf vegetable i.e removing the leaves from the stick of the vegetable	Bitter leaf	
1:30	BGS		Sleeping on the mat		
	MB		Still processing the vegetable removing the leaves from the stick of the vegetable	Bitter leaf	
	BB1		Crying and shouting at the mother		
	MDL1		Washing plates, bowls, cups and pots.		
1:35	MB		Still processing bitter leaves	Bitter leaf	
	BGS		Sleeping on the mat		
	BB1	BB2&BB3	Sharing ‘gumba’ (firm hard paste) among the other siblings	Maiwa	
1:40	MB		Still processing the bitter leaves	Bitter leaf	
	BGS		Sleeping on the mat alone		
1:45	BGS		Sleeping on the mat alone		
1:50	BGS		Sleeping on the mat alone		
	MB		Sitting beside the child on the mat while the child was sleeping		
	BB4		Feeding herself kunu	Kunun maiwa	
1:55	MB	BGS	Sitting on a stool and giving the baby water, carrying him on her laps	Water	

2:00	MB	BGS	Breastfeeding the baby	Breast milk	
	BB4	BB4	Crying and drinking kunu	Kunun maiwa	
2:05	MB	BGS	Breastfeeding the baby	Breast milk	
2:10	MB	BGS	Breastfeeding the baby	Breast milk	
	BB1	BGS	Playing with the child		
	BB2	BGS	Playing with the child		
2:15	MB	BGS	Walking and breastfeeding the child	Breast milk	
	BB4		Still crying and shouting at the mother		
	DL1		Washing black pots		
2:20	MB	BGS	Backing the baby		
2:25	MB	BGS	Backing the baby		
	BB4	BB4	Eating tuwo and vegetable soup	Tuwon maize and spinach soup	
	BB3	BB3	Eating tuwo and vegetable soup	Tuwon Maize and Spinach soup	
2:30	MB	BGS	Backing the baby		
		BGS	Playing on his mother back and stretching his hands as he wants to meet the siblings eating		
	BB4		Eating tuwo and spinach soup	Tuwo and spinach soup	
	BB3		Eating tuwo and spinach soup	Tuwo and	

				spinach soup	
	MB		Selling tamarind to a young girl within the neighborhood.		
2:35	MB	BGS	Backing the child		
2:40	MB	BGS	Backing the child		
	MB		Carrying a bucket of water on her head		
	BB3&BB4		Playing with sticks and stones		
2:45	MB	BGS	Backing the baby with a bucket of water on her head.		
2:50	MB	BGS	Backing the baby and standing		
	BGS		Playing with his tongue		
	DL2		Washing plates and bowls		
2:55	MB	BGS	Breastfeeding the child while standing	Breast milk	
3:00	MB	MDL2	Selling maiwa grains to her		
3:05	BGS		Lying on the bed alone while the mother is away.		
3:10	BGS		Lying on the bed and crying and the mother is still away.		
3:15	MB	BGS	Backing the baby while standing		
	MDL2		Processing maiwa grains to remove shafts	Maiwa grains	
	DL2		Washing plates and pots		
3:20	MB	BGS	Backing the baby and still standing.		
	MB		Setting the fire with firewood.		
	DL2		Washing of utensils, pots and bowls		
3:23	DL2		Putting pot on the fire		
	DL2		Adding water to the pot		

3:24					
3:25	MB	BGB	Backing the baby while standing		
	DL2		Adding firewood to the fire		
3:28	DL2		Adding washed dankaule to the pot of water	moringa	
3:33	MB	BGB	Backing the baby		
3:38	MB	BGB	Backing the baby		
	MB		Sweeping her corridor and bringing out dirty plates and bowls		
3:43	MB		Sweeping her compound and backing the baby.		
	BGB		Lying on a cane bed		
	BB4	BGB	Playing with the child		
	DL2		Washing another pot		
3:48	MB		Selling tamarind to a girl and later continues sweeping		
3:53	BGS		Lying on a cane bed		
	BB4	BGS	Playing with the child		
3:58	MB		Washing plates and bowls		
	BGS		Lying in a cane bed		
	BB4&BB3	BGS	Playing with the child		
	DL2		Checking th dankaule in the pot on fire.	moringa	
4:00	DL2		Setting another fire		
4:02	DL2		Putting a pot of water on the fire and adding more firewood		
4:04	DL2		Adding more water to the pot		
	MB		Washing plates and pots		

	BGS		Still playing with siblings		
4:14	BGS		Still playing with siblings		
	DL2		Setting the fire		
4:19	MB	BGS	Bathing the child		
4:24	MB	BB3&BB4	Bathing the other children		
4:29	MB	Bb3&bb4	Wearing new clothes for the children		She had to wear very new clothes for the children because of my presence.
	DL2		Adding firewood		
4:34	MB	BGB	Carrying the child		
4:39	BGB		Playing alone		
4:44	DL2		Checking the water which was already boiling		
4:49	BB1	BGS	Giving the child cooked dankaule	Moringa	
4:54	BGS		Eating cooked dankaule	moringa	
4:59			The water in the pot is boiling		
	MB	BGS	Breast feeding the child	Breast milk	
5:04	MB	BGS	Breast feeding the child	Breast milk	
5:09	MB	BGS	Breast feeding the child	Breast milk	
5:14	MB		Sieving water into the pot		
	BGS		Playing with stick		

5:19	BGS		Licking kuli-kuli	Kuli-kuli	
5:24	BGS		Fell from the cane bed and started crying		
	MB	BGS	Breastfeeding the baby	Breast milk	
		DL2	Setting the fire		
		DL1	Setting the fire also		
5:29	MB		Setting her own fire		
	BGS		Licking kuli-kuli	Kuli-kuli	
	DL2		Adding firewood		
5:34	BB1	BGS	Giving the child kunu	Kunun maiwa	
5:39	BGS		Licking kunu from the floor		
	MB		Washing rice in a basin		
	BGS		Crawling and playing around the house		
	DL2		Making paste for kunu by adding water to the fine sieved flour obtained and mixing properly.	Kunu maiwa	
5:04	MB		Adding rice to boiling water	Rice	
	BGS		The baby is crying		
	BB2	BGS	Carrying the baby while and he is still crying.		
	DL1		Setting the fire and placing pot on the fire		
5:54	BGS		The baby is still crying		
5:59	MB		Adding firewood to fire		
	DL2		Pouring hot water into a big bucket of paste to make kunu	Kunun maiwa	

6:04	MB		Adjusting the firewood		
		BGS	Taking him to his uncle's house		
	DL2		Adding small of the paste fetched from the kunu to make it thicker	Kunun maiwa	
6:09	MB		Adding water to the rice on the fire		
6:14	MB		Adding firewood		
	BGS		Playing with elder sister, but he is crying		
6:19	MB	BGS	Breastfeeding the child	Breast milk	
	DL2		Adding tamarind water to the kunu	Kunun maiwa	
	MB		Adding firewood to the fire		
6:24	BB3	BGS	Feeding the child kosan rogo	Cassava bean	
6:29			Eating kosan rogo		
6:34	MB		Checking rice on the fire		
	BGS		Playing and crawling around		
6:39	MB		Bringing the pot down from the fire		
	DL		Adding sugar and mixing the kunu again		
	BGS		Playing with stones		

FIELD NOTES

Data collector: The mother of the child was asked if she has administered the MNP to the child today

Mother's response: '*I administer one sachet to the child everyday but I haven't done that today because it was unfortunate that the leftover kunun maiwa has become fermented. I decided to wait till evening after a new kunu has been prepared.*

Husband's: '*she has been administering the MNP ever since it was brought'*

Primary Questions (Complete after you complete data collection):

From what you have seen observing THIS HOUSEHOLD, please answer the following questions in detail:

Question	Write your detailed response here
1. In this household, what would be the best way(s) to give nutritional supplements such as an MNP to young children to ensure compliance?<br><br>Why?	The best way to give the MNP in this is through then mother.<br><br>Because the child is always with the mother, she carries her along all the time. She also stays beside the child even he is sleeping or playing.
2. In this household, who is favored during meal	I was opportune to see the meal sharing during the day when few

preparation or feeding?<br><br>How is this favoritism expressed?	children were eating. I realized that it is shared amongst peer group and the younger children are more favored.<br><br>The younger ones are served first and to their satisfaction and the little ones are mostly fed by their mothers.
3. How could we promote the MNP supplement for this family that would attract their attention?<br><br>Who would they listen to?	The MNP should be given to all the mothers with children of the required age in the household since it is an extended household.<br><br>They will listen to their husbands and their father in-law. Also the mother-in-law because, I observed she is well respected by her daughter in-laws although she is always by her side. Also they are answerable to her orders and take commands from her.
4. In this household, what would be the best ways to promote the MNP but limit how much it would be shared?<br><br>Why?	There should be proper sensitization about the MNP; also its mode of administration should be properly stated to the caregivers and husbands/mother in-laws. Also food sharing among peer group should either be discouraged or they should be told to take the small portion and add the MNP first, after the child finishes, he can be fed by the other siblings.<br><br>Because if it is clearly stated and its importance stated also, they will consider it as medicine and will make sure it is only given to the child.
5. What other issues and	The major concern or challenge that maybe facing this family is that

concerns did you see with this family that are important for us to consider with regard to introduction of an MNP?<br><br>Why?	it is an extended family with 2 mother in-laws and 5 daughter in-laws.<br><br>Because all the daughter in-laws are caregiver and they all have to be issued the MNP to overcome the issue of discouraging other caregivers within the household from using it because of envy.
6. Based on your experience observing this child today, do you believe reactivity negatively impacted your ability to see the food-related behaviors of the child?<br><br>Please explain your answer in detail.	NO<br><br>Because the child just had a few meal intake throughout my stay because he is just 7months and is practicing complementary feeding little by little.
